# Exposure to Titanium Dioxide Nanoparticles During Pregnancy Changed Maternal Gut Microbiota and Increased Blood Glucose of Rat

**DOI:** 10.1186/s11671-018-2834-5

**Published:** 2019-01-17

**Authors:** Zhilei Mao, Yaqi Li, Tianyu Dong, Lina Zhang, Yuqing Zhang, Shushu Li, Haiting Hu, Caifeng Sun, Yankai Xia

**Affiliations:** 1 Changzhou Maternity and Child Health Care Hospital affiliated to Nanjing Medical University, Changzhou, 213003 Jiangsu China; 2 State Key Laboratory of Reproductive Medicine, Institute of Toxicology, Nanjing Medical University, 101 Longmian Road, Nanjing, 211100 China; 3 Key Laboratory of Modern Toxicology of Ministry of Education, School of Public Health, Nanjing Medical University, Nanjing, 211100 China

**Keywords:** TiO_2_ NPs, Gut microbiota, Pregnancy exposure, Increased fasting blood glucose

## Abstract

Titanium dioxide nanoparticles (TiO_2_ NPs) were used worldwide for decades, and pregnant women are unable to avoid exposing to them. Studies revealed that TiO_2_ NPs could kill many kinds of bacteria, but whether they would affect the composition of gut microbiota, especially during pregnancy, was seldom reported. And, what adverse effects may be brought to pregnant females was also unknown. In this study, we established the prenatal exposure model of rats to explore the effects of TiO_2_ NPs on gut microbiota. We observed an increasing trend, but not a significant change of alpha-diversity among control and exposure groups at gestation day (GD) 10 and GD 17 during normal pregnancy process. Each different time point had unique gut microbiota operational taxonomic units (OTUs) characteristics. The abundance of Ellin6075 decreased at GD 10 and GD 17, Clostridiales increased at GD 10, and Dehalobacteriaceae decreased at GD 17 after TiO_2_ NPs exposure. Further phylogenetic investigation of communities by reconstruction of unobserved states (PICRUSt) prediction indicated that the type 2 diabetes mellitus related genes were enhanced, and taurine metabolism was weakened at the second-trimester. Further study showed that the rats’ fasting blood glucose levels significantly increased at GD 10 (*P* < 0.05) and GD 17 (*P* < 0.01) after exposure. Our study pointed out that TiO_2_ NPs induced the alteration of gut microbiota during pregnancy and increased the fasting blood glucose of pregnant rats, which might increase the potential risk of gestational diabetes of pregnant women.

## Introduction

Titanium dioxide nanoparticle (TiO_2_ NP) is one of the most widely used nanomaterial, and can be easily found in sunscreen, paint, ink, and foods [1, 2]. They can easily be released and enter human body during the usage of commercial products. Notably, the pregnant women cannot avoid exposing to them. Animal studies had shown that the ovarian and reproductive system dysfunction were observed [3], and monoaminergic neurotransmitters were also impaired [4] when adult female mice were exposed to TiO_2_ NPs. Further, pregnancy complications and adverse birth outcomes were also observed after pregnant mice exposed to TiO_2_ NPs [5]. All studies above indicated that TiO_2_ NPs were harmful to adult female animals, as well as the pregnant females, but the mechanisms were not fully understood. So the relative studies need to be carried out for the safety evaluation of TiO_2_ NPs.

TiO_2_ NP is used as a kind of powerful antibacterial agent; they can kill many types of bacteria, including *Staphylococcus aureus*, Salmonella, *Streptococcus mutans*, and so on [6]. The antibacterial effects were nonselective actually, while most of the current studies mainly focus on their effects on killing harmful bacteria, few reported whether TiO_2_ NPs would kill probiotics or other symbiotic bacteria and bring adverse effects to human beings. Studies about whether TiO_2_ NPs would change normal composition of gut microbiota and cause disadvantages to pregnant females were also lacking; therefore, we carried out this study from the perspective of gut microbiota.

Recently, more and more researches showed that gut microbiota were closely related with human disease including type 2 diabetes [7] and obesity [8]. Probiotics could affect the metabolic of pregnant women with gestational diabetes [9], and change the methylation of diabetes-associated genes in fetuses [10]. Studies reported that the plasma glucose level increased when adult mice were exposed to TiO_2_ NPs for 12 weeks [11]. Whether the blood glucose of pregnant females would increase after exposure and whether the exposure period would shorten were not reported.

All the studies mentioned above suggested that TiO_2_ NPs may affect gut microbiota and increase the plasma glucose level, but no direct evidence proved the linkage between gut microbiota and maternal blood glucose level, and the mechanisms were also not clear. Previous studies mainly focus on adult animal studies, and the effects of TiO_2_ NPs on pregnant females were merely studied from the perspective of gut microbiota. In this study, we established the pregnancy exposure model of rat to explore whether the maternal gut microbiota would change and how they change after the pregnant females exposed to TiO_2_ NPs, and we tried to answer the issue that what adverse effects would be brought to the pregnant females by gut microbiota changes after TiO_2_ NPs exposure. Our study raised the concerns about the safety of TiO_2_ NPs to the pregnant women and we revealed the potential mechanisms.

## Materials and Methods

### Study Design

**Fig. 1:**
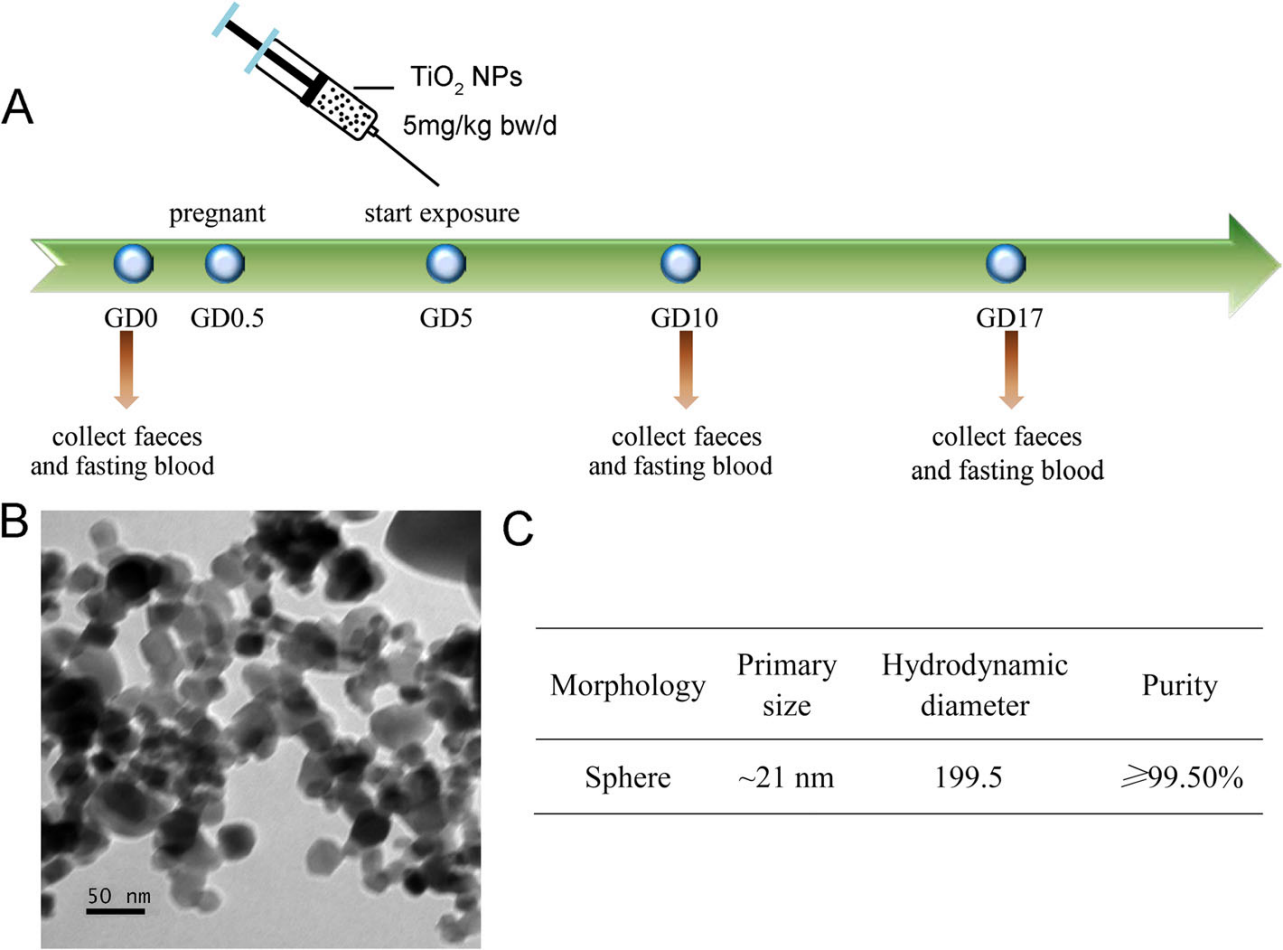
**a** The experimental design of this study. **b** The TEM images of TiO_2_ NPs, bar = 50 nm. **c** Main characteristics of TiO_2_ NPs measured or reported by manufacturer were presented

On the basis of a study carried out by Weir, A. and his colleagues in human beings [12], the exposure route and exposure dose in rats were determined. The female rats were daily gavage administrated with 5 mg/kg bw/day of TiO_2_ NPs from the 5th to 18th day after pregnancy, and the progress was shown in Fig. 1a. Each rat was weighed before oral exposure, and 0.5% of the methylcellulose was given as vehicle control.

### Animals

Animal studies were performed with the permission of the ethics committee. Sprague-Dawley (SD) rats were purchased from Beijing Vital River Laboratory Animal Technology Co., Ltd. Female rats (*n* = 8, 12 weeks old) were separated from male rats (*n* = 8, 14 weeks old), and rats of the same gender were kept in a large cage. All rats were housed in a temperature- (22 ± 2 °C) and humidity-controlled (40–60%) condition, with a 12 h light/dark cycle for 1 week rest. Then the female rats were randomly divided into control group (*n* = 4) and exposure group (*n* = 4), and mated with males at a 1:1 ratio in individual cages. Vaginal plug were observed every morning and the presence of vaginal plug confirmed pregnancy and recorded as gestation day 0.5 (GD 0.5), and the pregnant rats were raised in separated cages.

### TiO_2_ NPs Preparation and Administration

TiO_2_ NP is a commercial product purchased from Sigma-Aldrich (13463-67-7). The stock solution of TiO_2_ NPs were dissolved in methylcellulose (0.5%) at the concentration of 5 mg/ml according to a previous study [13], and sonicated for 30 min (100 W). The hydrodynamic diameter of TiO_2_ NPs in methylcellulose was measured with dynamic light scattering (DLS).

### Feces Collection and Fecal Total DNA Preparation

The feces of each rat were collected at GD 0 (before mating), GD 10, and GD 17 with the process of pregnancy, respectively. The feces were stored at − 80 °C before bacterial diversity were analyzed. Fecal total DNA was extracted using a Power Soil DNA kit (Mo Bio Laboratories, Carlsbad, California, USA) according to the manufacturer’s protocol. And the DNA concentrations were measured by NanoDrop spectrophotometer (NanoDrop™ 2000/2000C, USA).

### 16S rRNA Gene Sequencing and Data Analysis

Bacterial sequencing of 16S rRNA genes was performed with the Illumina MiSeq platform (Hangzhou Guhe Information and Technology Co., Ltd., Zhejiang, China). The V3 and V4 regions of bacterial 16S rRNA were amplified with specific primers as previously described [14]. And the DNAs were subjected for Illumina MiSeq sequencing after amplified and purified. The sequencing data were processed using quantitative insights into microbial ecology (QIIME) according to previous studies [15]. Data was read and merged from original DNA fragments, and the read lengths were between 400 and 500 bp. Chimeric sequences was further examined using QIIME if occurs.

### Blood Sample Collection and Blood Glucose Determination

The fasting venous blood of all female rats was also collected accordingly when feces were collected. The blood samples were collected from caudal vein in the morning after 12 h of starvation at GD 0, GD 10, and GD 17, respectively. Then the fasting blood glucose levels were immediately determined with Roche ACCU-CHEK® Performa meter according to the manufacturer’s protocol after collection.

### Statistical Analysis

Statistical analysis were performed with Graphpad Prism 6; all data about the diversity of bacterium were presented with box plots as Mean ± SE, and significance of among all groups were examined by one-way ANOVA followed by Dunnett’s multiple comparison test. *P* < 0.05 was considered as statistically significant.

## Results and Discussion

### Characteristics of TiO_2_ NPs

The main characteristics of TiO_2_ NPs were measured and presented before animal studies. Figure 1b showed a visual field of TiO_2_ NPs under transmission electronic microscope. The morphology of TiO_2_ NPs was nearly sphere with a primary diameter of about 21 nm. The average hydrodynamic diameter was about 199.5 nm in methylcellulose solution (Fig. 1c). The purity of TiO_2_ NPs is *≥* 99.5%, and the surface area is 35–65 m^2^/g according to the manufacturer’s report. Recent studies reported that both nano- and fine grade TiO_2_ could increase the blood glucose level of adult animals after oral exposure [11, 16], and whether the blood glucose of the pregnant females would be affected was unknown. To make this question and underlying mechanisms clear, we established the pregnancy rat exposure model to evaluate the toxicity of TiO_2_ NPs and to probe the harms to pregnant rats.

Most TiO_2_ particles in products are with primary size of mainly ranging from 60 to 300 nm, minority (~ 20%) was < 100 nm [17], while recent study showed that the amount of TiO_2_ NPs in some food products is much larger than we known (~ 90%), for instance, chewing gum [18]. As known, smaller nanoparticles had higher toxicity [19, 20], and the females were more sensitive to harmful substrates during pregnancy, so the minority part of TiO_2_ NPs may bring nonnegligible effects to pregnant females than the majority fine particles. In this study, we exposed the pregnant rat model to nanosized TiO_2_ (~ 21 nm) to study the potential risks of TiO_2_ NPs to pregnant women.

### Bacteria Diversity Changes During Normal Pregnancy

**Fig. 2:**
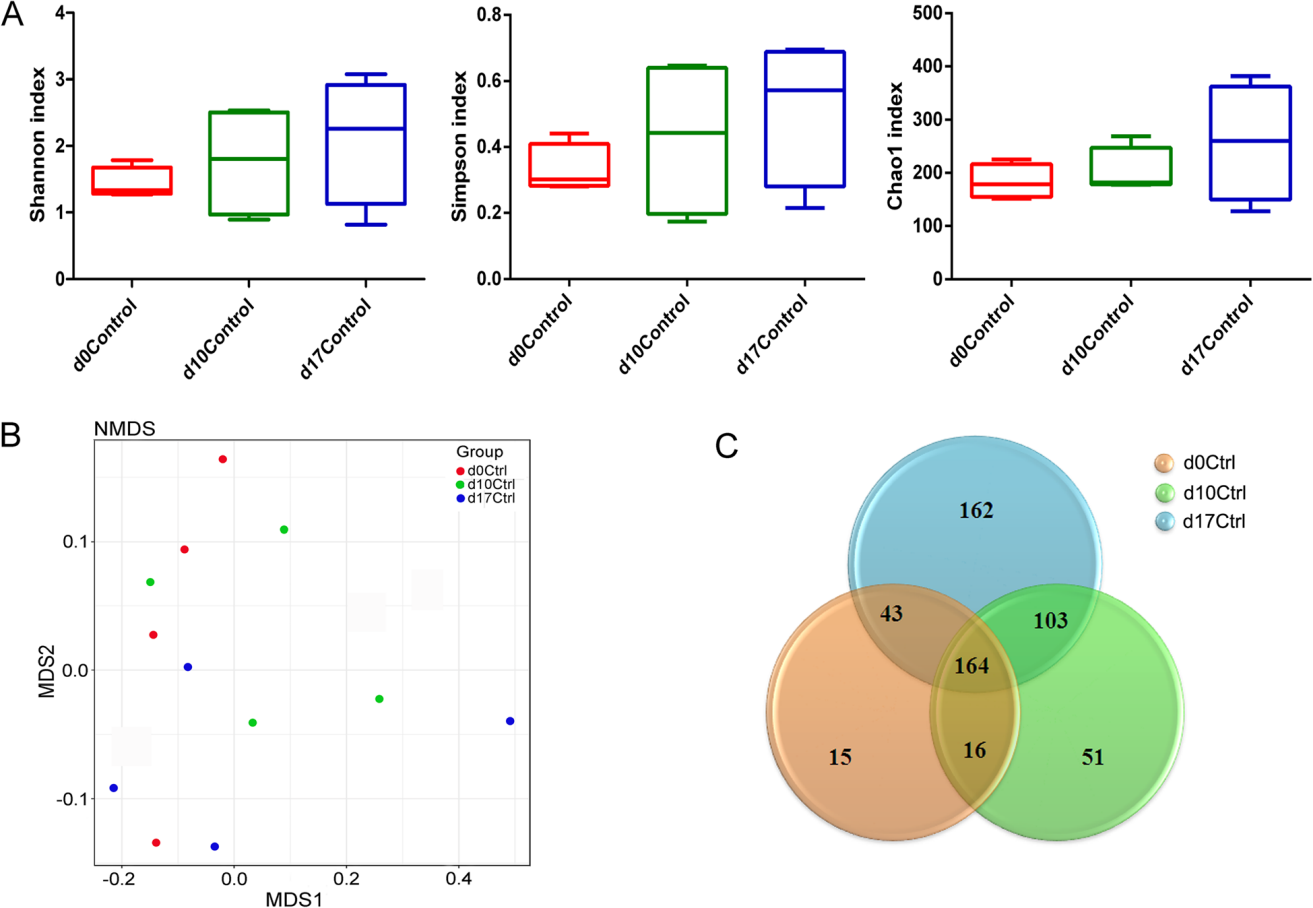
Changes in the characteristics of gut microbiota in control (unexposed) rats in the course of a normal pregnancy. **a** Alpha-diversity of gut microbiome revealed by Shannon, Simpson, and Chao1 index. **b** The beta-diversity was revealed by non-metric multi-dimensional scaling (NMDS) analysis. **c** The unique and sharing operational taxonomic units (OTUs) numbers were presented in Venn diagram. D0 Ctrl means rats in control group before mating, d10 Ctrl and d17 Ctrl means the control rats in GD 10 and GD 17, respectively

During gestation, pregnant females become more sensitive to physical and chemical exposure; in order to decrease the effects of manual operation to the implantation of fertilized egg, the 5th day was chosen as the first day of exposure when the blastulas had finished implantation. GD 17 is the last day before delivery and GD 10 is the midterm of pregnancy. The normal dynamics of gut microbiome during pregnancy was examined using fecal samples from three time points of control groups (GD 0, GD 10, and GD 17). We observed the alpha-diversity of gut microbiome over time by computing Shannon, Simpson, and Chao1 indexes, but the difference was not significant (Fig. 2a). Based on non-metric multi-dimensional scaling (NMDS) analysis, no marked difference was also found in samples from different time points (Fig. 2b), which was consistent with previous studies [21, 22]. The Venn diagram (Fig. 2c) showed the shared and specific operational taxonomic units (OTUs) in samples of different time points, and the shared OTUs of three time points (GD 0, GD 10, GD 17) in control groups was 164; these results indicated that the number of specific OTUs was increased with time during pregnancy. Our results showed that the gut microbiota revealed no significant change during normal pregnancy, and the changes will not bring adverse effects and are even beneficial to maternal. Our results suggested that the gut microbiota change might be the result of pregnancy process, which might be caused by hormonal changes of pregnant females [23], similar as vaginal flora changes during pregnancy [24]. Also, it might be a precondition for normal pregnancy.

### Bacteria Diversity Changes After Exposed to TiO_2_ NPs During Pregnancy

**Fig. 3:**
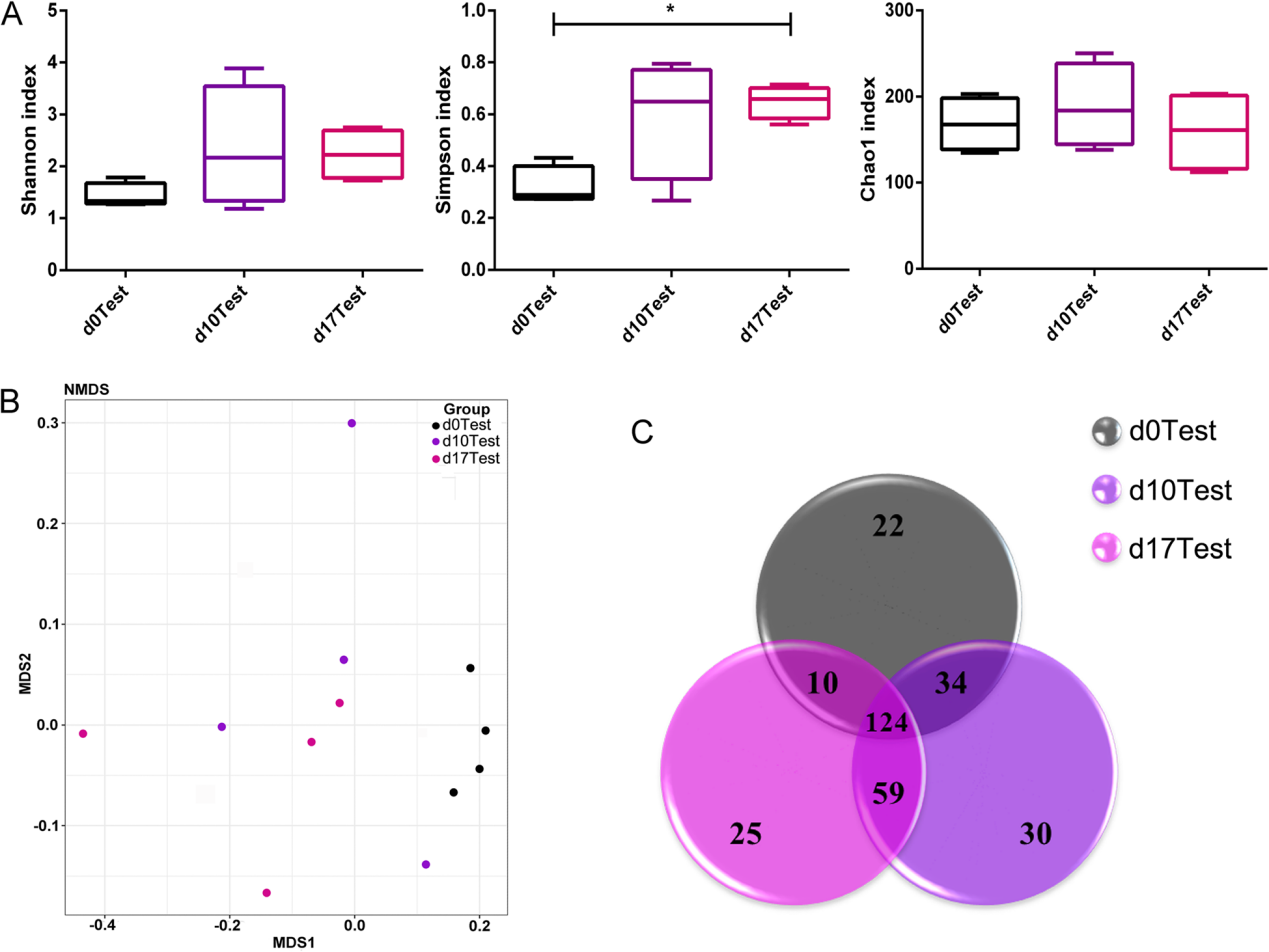
Changes in the characteristics of gut microbiota in exposed rats in the course of pregnancy. **a** The alpha-diversity of gut microbiome revealed by Shannon, Simpson, and Chao1 index. **b** The beta-diversity was revealed by NMDS analysis. **c** The unique and sharing OTUs numbers were presented in Venn diagram. D0 Test means samples collected from rats before exposure to TiO_2_ NPs, d10 Test, and d17 Test means samples collected from exposed rats at GD 10 and GD 17, respectively

Studies indicated that gut microbiota is crucial for the maintenance of normal immunity situation [25]; a natural change of gut microbiota during normal pregnancy may regulate the immunity system to accept the implantation of fertilized eggs [26]. Meanwhile, the natural alteration of gut microbiota during normal pregnancy might also help the pregnant women adapt to the metabolic changes during gestation. Once the alteration of gut microbiota exceeded a “proper degree,” adverse pregnancy outcome may be brought. So we analyzed the microbiota changes after TiO_2_ NPs exposure in the following part. The effects of TiO_2_ NPs on bacteria diversity during pregnancy were evaluated by analyzing alpha-diversity and beta-diversity at GD 0, GD10, and GD17 after females were exposed to nanoparticles. The results showed that the alpha-diversity showed an increasing trend in Shannon, and a significant change in Simpson index (*P* < 0.05) when comparing to normal pregnancy, but no difference in Chao1 (Fig. 3a). The NMDS analysis (Fig. 3b) also showed no significant difference as it is in normal pregnancy, but after exposure to TiO_2_ NPs, the specific OTUs in samples decreased at middle and late pregnancy (Fig. 3c). During normal pregnancy, the diversity of gut microbiota had no obvious change, but we observed an increasing trend of bacteria diversity in maternal feces after female mice were exposed to TiO_2_ NPs during pregnancy, which might be due to TiO_2_ NPs being a highly efficient antibacterial agent, and could kill many kinds of bacteria; they inhibited the dominant bacteria in gut and the originally suppressed bacteria could reproduce under this condition. Studies indicated that gut microbiota was associated with many diseases, including diabetes, obesity, hypertension [27], and cancer [28]; the linkage between gut microbiota and gestational diabetes had also been confirmed [29]. The reason why no significant change among GD 0, GD10, and GD17 was observed after exposure may be that TiO_2_ NPs was “relatively safe” or the microbiota changes induced by TiO_2_ NPs exposure may be covered by the pregnant-related microbiota changes.

### Gut Microbiota Changes in Second Trimester After Exposed to TiO_2_ NPs

**Fig. 4:**
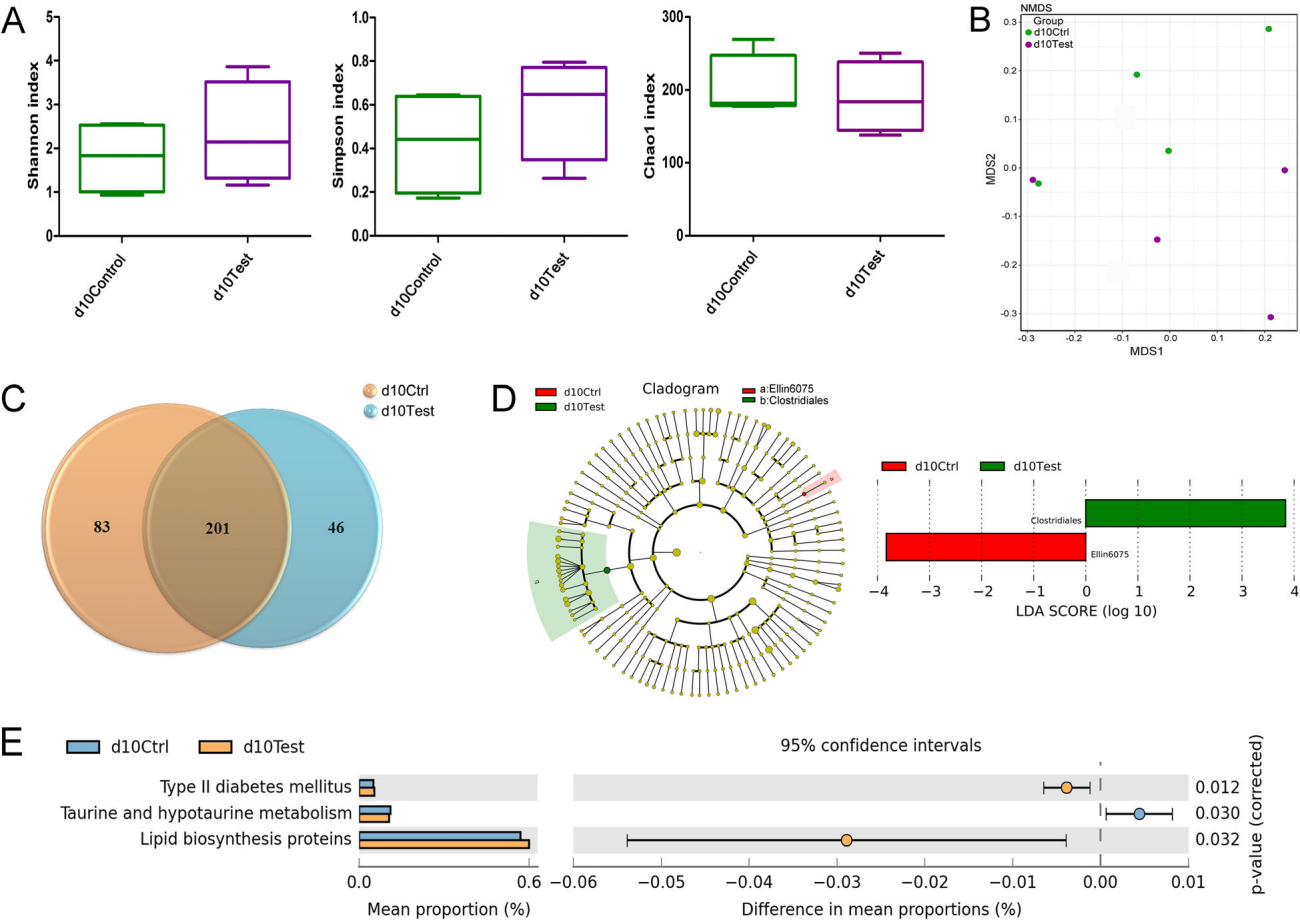
Changes in the characteristics of gut microbiota between control rats and TiO_2_ NP exposed rats at GD 10. **a**, **b** Alpha- and beta-diversity of gut microbiome were presented as Shannon, Simpson, and Chao1 index, as well as NMDS analysis. **c** Venn diagram showed the OTUs characteristics. **d**, **e** Dominant biomarkers and related gene functions were found by Lefse and phylogenetic investigation of communities by reconstruction of unobserved states (PICRUSt) prediction, respectively. D10 Ctrl means the control rats at GD 10, d10 Test means the exposure rats in GD 10

To exclude the effects of pregnancy and further find out the independent effects of TiO_2_ NPs on gut microbiota, we compared the differences of gut microbiome between control group and treatment group using samples collected from second trimester (GD 10). No significant difference of alpha-diversity was found according to Shannon, Simpson, and Chao1 indexes (Fig. 4a). A remarkable distinction was observed between the two groups based on NMDS analysis (Fig. 4b). Figure 4c showed that exposure of TiO_2_ NPs led to the changes of some specific OTUs in treatment group compared with the control (Venn). These results showed that TiO_2_ NPs were relatively safe and will not induce obvious dysbacteriosis. But the flora composition, namely the abundance of specific genus, changed in the second trimester and late pregnancy stage respectively. To further find out the potential risks of the changes that happed during pregnancy and explore what adverse effects may be brought, we identified the functional changes of the gut microbiota with bioinformatics. The results showed that two dominant biomarkers, Ellin6075 and Clostridiales, were found by LefSe analysis (linear discriminant analysis (LDA) > 2). Abundance of Ellin6075 was decreased and Clostridiales was increased after TiO_2_ NPs exposure respectively (Fig. 4d). Ellin6075 was isolated from an Australia farm, but little information was available regarding its phenotypic traits or functions, so their effects on pregnancy need further investigation. Yan and his colleagues showed that Clostridium significantly increased in obesity SD rats [30], which were consistent with our finding that Clostridiales co-existed with high level of blood glucose. To reveal the effects of gut microbiota change on pregnancy, we predicted the gene differences in fecal samples using phylogenetic investigation of communities by reconstruction of unobserved states (PICRUSt) (Fig. 4e), and found that genes about type 2 diabetes mellitus related function and lipid biosynthesis proteins were strengthened in treatment group, while taurine and hypotaurine metabolism was weakened. Researchers had demonstrated that gut microbiota could generate short-chain fatty acid, including acetic acid, propionic acid, and regulate the host blood glucose in turn [31]. And the change of taurine and hypotaurine were also in accordance with the fact that taurine could downregulate the maternal blood glucose concentrations [32].

### Gut Microbiota Changes in Late Pregnancy After Exposed to TiO_2_ NPs

**Fig. 5:**
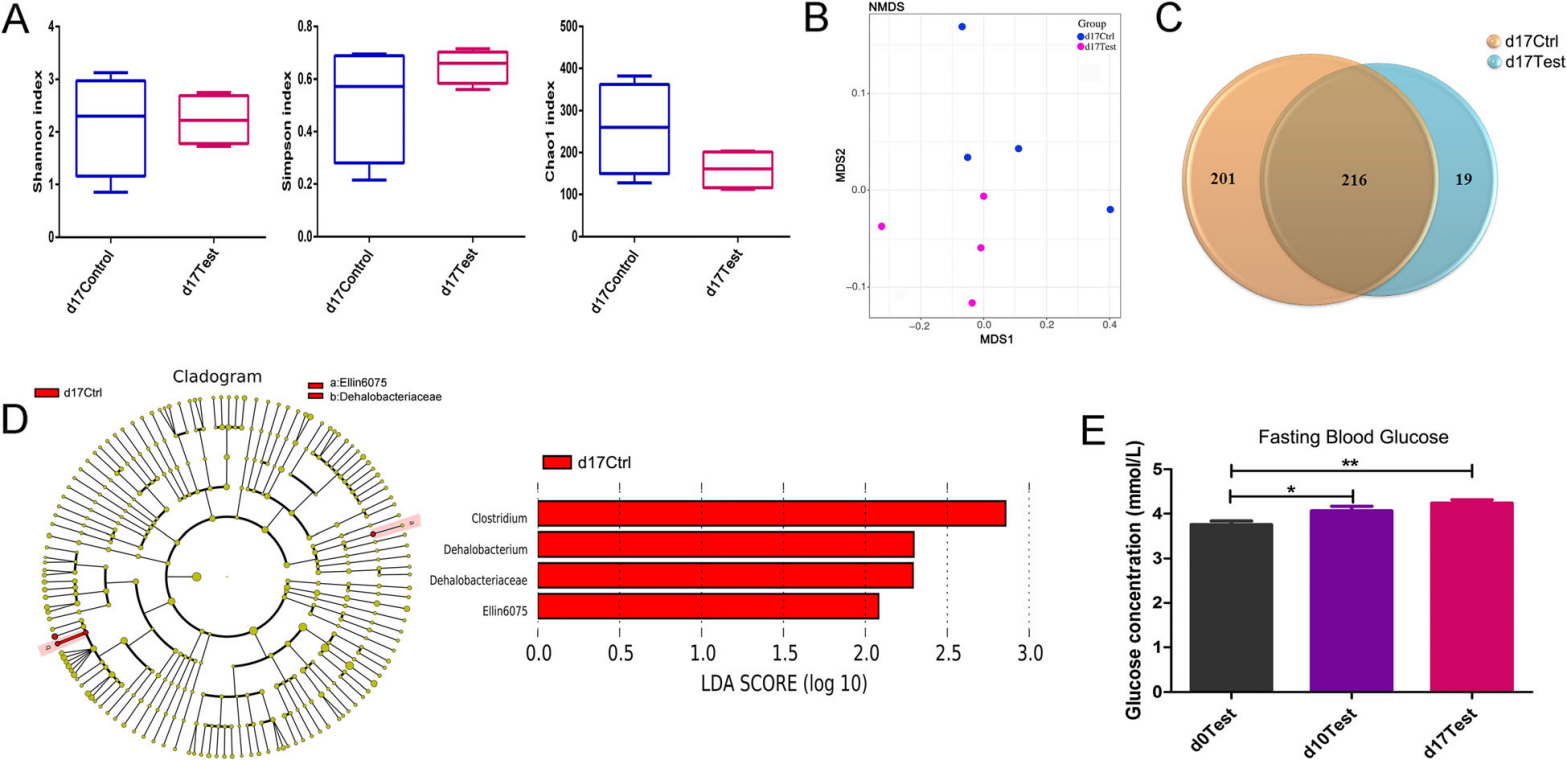
Changes in the characteristics of gut microbiota between control rats and TiO_2_ NP exposed rats at GD 17. **a** Shannon, Simpson, and Chao1 index which presented alpha-diversity were compared between two groups. **b** The beta-diversity was revealed by NMDS analysis. **c** The Venn diagram showing the unique and sharing OTUs in two groups. **d** Lefse found the candidate biomarkers (LDA > 2) and the differences of gene functions were predicted. **e** The fasting blood glucose levels of rats were measured at GD 0, GD 10, and GD 17 after exposed to TiO_2_ NPs. D17 Ctrl and d17 Test means the control and exposed rats on the 17th day of pregnancy, respectively

Gut microbiome of late pregnancy was examined by fecal samples collected at GD17. No significant difference was found in alpha-diversity (Fig. 5a). These samples were districted significantly by control and treatment group in the NMDS model (Fig. 5b). As shown in Fig. 5c, decreased number of observed OTUs was found in the treatment group. We also used Lefse to identify potential biomarkers. Notably, as shown in Fig. 5d, abundance of Ellin6075 persisted decreased in the treatment group during late pregnancy (LDA > 2), and the abundance of Dehalobacteriaceae was decreased by exposure to TiO_2_ NPs as well (LDA > 2). In this stage, the diabetes mellitus-related gene changes was not observed, which suggested that the second trimester, instead of the late trimester, was the sensitive window for TiO_2_ NPs to increase the maternal blood glucose. And the result was in agreement with our clinical recognition that, our doctors carried out the oral glucose tolerance test (OGTT), a common diagnosis of human gestational diabetes, to screen gestational diabetes in pregnant women at the second-trimester (about 26th week in pregnant women). The results showed that the fasting blood glucose had increased on GD 10 after the pregnant rats exposed to TiO_2_ NPs, and is prior to a previous result reported (~ 12 weeks) in adult animals [11], which proved the fact that pregnant females were more sensitive than adults.

### The Effects of TiO_2_ NPs on Blood Glucose After Prenatal Exposure

In order to prove the results of PICRUSt prediction, we measured the rats’ fasting blood glucose at GD10 and GD17, respectively. After the pregnant rats were exposed to TiO_2_ NPs for 12 days (GD5–GD17), the fasting blood glucose levels were measured. As shown in Fig. 5e, comparing with control group, the rats’ fasting glucose levels increased significantly at both GD10 (*P* < 0.05) and GD17 (*P* < 0.01) after exposed to TiO_2_ NPs, which was in accordance with the previous reports that TiO_2_ NPs could increase the blood glucose level of adult animals [11, 33]. But the increment of value between control group and GD 17 was relatively small (~ 0.5 mM), and did not reach the standard of gestational diabetes [34]. The results suggested that, maternal solitary exposed to TiO_2_ NPs during pregnant is not sufficient to induce gestational diabetes, but the increased blood glucose may bring adverse effects to the pregnant females and their offspring. And it was reported that maternal exposed to higher blood glucose during pregnancy might increase the risks of obesity and abnormal glucose tolerance of fetuses [35], which also reminded us that TiO_2_ NPs may bring potential risks to offspring.

## Conclusion

Our studies indicated that prenatal exposure of TiO_2_ NPs could increase maternal fasting blood glucose levels, and the gut microbiota alterations might be the underlying mechanism. And we draw the conclusion that TiO_2_ NPs might increase the risk of gestational diabetes of human pregnant women, which should arouse our attentions.

## Abbreviations

DLS: Dynamic light scattering
GD: Gestation day
LDA: Linear discriminant analysis
NMDS: Non-metric multi-dimensional scaling
OGTT: Oral glucose tolerance test
OTUs: Operational taxonomic units
PICRUSt: Phylogenetic investigation of communities by reconstruction of unobserved states
QIIME: Quantitative insights into microbial ecology
SD: Sprague-Dawley
TiO_2_ NPs: Titanium dioxide nanoparticles
